# Thin-layer modeling, drying parameters, and techno-enviro-economic analysis of a solar dried salted tilapia fish fillets

**DOI:** 10.1038/s41598-025-87807-w

**Published:** 2025-02-11

**Authors:** Tarek Hussien M. Ghanem, Loai S. Nsasrat, Omar Shahat Younis, Khaled A. Metwally, Ali Salem, Zoltan Orban, Mohamed Hamdy Eid, Hany S. El-Mesery, Abdalla Zain Eldin, Khaled Mohamad Elmolakab, Samy F. Mahmoud, Abdallah Elshawadfy Elwakeel

**Affiliations:** 1 Agricultural Products Processing Engineering Department, Faculty of Agricultural Engineering, Al Azhar University, Cairo, Egypt; 2 Electrical Power Engineering Department, Faculty of Engineering, Aswan University, Aswan, 81528 Egypt; 3 Food Manufacturing Engineering and Packaging Department, Food Technology Research Institute, Agriculture Research Center, Giza, Egypt; 4 Soil and Water Sciences Department, Faculty of Technology and Development, Zagazig University, Zagazig, 44519 Egypt; 5 Civil Engineering Department, Faculty of Engineering, Minia University, Minia 61111, Egypt; 6 Structural Diagnostics and Analysis Research Group, Faculty of Engineering and Information Technology, University of Pécs, Pécs 7622, Hungary; 7 Institute of Environmental Management, Faculty of Earth Science, University of Miskolc, Miskolc-Egyetemváros, 3515 Hungary; 8 Geology Department, Faculty of Science, Beni-Suef University, Beni-Suef, 65211 Egypt; 9 School of Energy and Power Engineering, Jiangsu University, Zhenjiang, 212013 China; 10 Agricultural Engineering Research Institute, Agricultural Research Center, Giza, 12611 Dokki Egypt; 11 Agricultural and Biosystems Engineering Department, Faculty of Agriculture, Alexandria University, Alexandria, Egypt; 12 Agricultural Engineering Department, Faculty of Agriculture and Natural Resources, Aswan University, Aswan, Egypt; 13 Department of Biotechnology, College of Science, Taif University, Taif City, Saudi Arabia

**Keywords:** Fish, Solar energy, Solar drying, Carbon footprint, Effective moisture diffusivity greenhouse gas, Mathematical modeling, Environmental sciences, Energy storage, Agroecology, Environmental economics

## Abstract

This study focused on the development of an indirect forced solar dryer that incorporates a three-sided flat plate solar collector (TSFPSC) specifically designed for increasing thermal efficiency, and the system used for drying salted tilapia fish fillets (STFF). The investigation analyzed three fillet thicknesses—4 mm, 8 mm, and 12 mm, employing both open sun drying (OSD) and the developed solar dryer (DSD), with a constant airspeed of 0.5 m/s. The research additionally developed thin-layer drying models (TLDM), assessed drying parameters, and performed an extensive techno-enviro-economic analysis. Results showed that the initial and final moisture content (MC) (w.b. %) of the STFF were 74.83 and 18.84%, respectively, and reached the equilibrium MC after 16–20.5 h for the DSD and 30–36 h for the OSD, which means the drying time reduced by about 53.3%, and 61.11% compared with the OSD. This reduction in drying time demonstrates the effectiveness of the developed solar dryer. The effective moisture diffusivity (EMD) of different STFF samples at both drying systems were 0.51 × 10^–10^ to 9.16 × 10^–10^ m^2^/s. In addition, all eleven basic TLDM were applied to predict the drying behavior of STFF during the drying process, while the combined Two-Term and Page model had the best fitting for the OSD system, and the modified Midilli II model and combined Two-Term and Page model had the best fitting for the DSD system. In terms of economic analysis, the annual capital and investment costs were calculated to be $22.458 and $21.334, respectively. Additionally, the environmental analysis indicated an energy payback (EP) period of 1.59 years, with a net CO_2_ mitigation of 14 tons realized over the operational lifetime of the DSD.

## Introduction

Aquatic products serve as crucial sources of high-quality protein in the human diet. However, they are very perishable and have a limited shelf life^1,2^. Each year, around 89% of the whole fisheries’ output is used for direct consumption by humans, with only about 45% of these fish eaten in their live and fresh state^3^. The FAO of the United Nations has endorsed tilapia fish as a freshwater breeding option because of its quick development, huge size, high protein content, and low-fat content. It is the second most frequent aquatic product^4^. However, in several developing nations, as much as 50% of the fish caught in these countries is discarded or unused^5^. This is because fish is often harvested with a high MC of 5 kg water per kilogram of dry matter (d.b.)^6^. If the fish is not preserved, it will quickly perish within 24 h, even without any external contamination^7^. Furthermore, most artisanal fish landing locations are often situated at a considerable distance from areas of consumption and market hubs. Conservation of these fish species will bolster the income of artisanal fishermen and contribute to the improvement of food security. Furthermore, the living conditions of the fishing community would be enhanced, leading to an increase in national revenue. Hence, it is essential to implement strategies to reduce waste and safeguard the fish sector^8^.

The process of drying fish is crucial as it prevents the formation of bacteria and mold by deactivating enzymes and eliminating the MC required for their proliferation^4,9,10^. Where dried fish is a significant marine product that is exported by many nations around the world. The drying of fish is mostly conducted using conventional methods, such as the OSD technique^1^. The most common method of fish drying throughout history has been OSD, where the fish is dried in the open air^11^. This method presents a risk due to many contaminated resources from sand-laden winds, rain, animals, harmful insects, dust, and soil. The OSD process is difficult because air temperature and relative humidity cannot be controlled daily, and it also takes too long^12–14^. So, the OAD method typically fails to meet the necessary quality standards, and the items cannot be sold on the global market^15^. Various contemporary drying methods have been used for drying aquatic items. For instance, Cao et al.^2^ utilized hot-air-aided radio frequency heating to dry tilapia fish fillets. The findings showed that the drying rate (DR) increased by roughly 1.1–1.4 times. Chukwu^16^, conducted research on the impact of different methods for drying tilapia fish fillets and catfish on the chemical composition. Pathare^17^ examined the characteristics of chela and prawn fish using an OSD at different temperatures and relative humidity. Boudhrioua et al.^18^ found that the EMD of sardine fillets ranged between 3.0–3.80 × 10^−9^ m^2^/s. Toujani et al.^19^ identified the suitable TLDM for the air-drying silverside fish and concluded that the Midilli and two-term models exhibited the highest level of accuracy. Understanding the basic transport mechanism of materials throughout the TLDM process is crucial because it lays the foundation for accurately simulating or scaling the whole process in order to optimize or regulate operational conditions^20^. Researchers have shown that neglecting the mathematical parts of drying kinetics and depending just on experimental drying approaches may have substantial consequences for the effectiveness of solar dryer (SD), leading to higher production costs and worse quality of the final product. Therefore, a highly efficient model is crucial for process design, energy integration, optimization, and control. Utilizing mathematical models to assess the process of drying agricultural products is crucial in this context^21,22^.

As well as, several research studies have investigated the economic performance of the SD, such as: Sajith and Muraleedharan^23^ performed an economic evaluation of a hybrid PV/solar drying system using an electric resistance wire. Elhage et al.^24^ conducted a comprehensive evaluation of OSD, including both their enviro-economic aspects. Hicham El Ferouali et al.^25^ conducted research on optimizing a hybrid solar-electric drying system designed explicitly for emerging nations. Also, the study done by Ndukwu et al.^26^ investigated the energetic sustainability and economic evaluation of a hybrid biomass-assisted SD that utilized a copper-heat exchanger. Metwally et al.^20^ investigate the environmental and economic attributes of a smart, automated sun drying system that is combined with a PV system for the purpose of drying date fruit. Habibi et al.^27^ performed a study on the energy and economic factors related to the sun-drying process of tomato slices. Ekka et al.^28^ offered support and examined the efficiency and techno-enviro-economic factors of a forced convection mixed mode sun drying system. Sharshir et al.^29^ investigated the thermo-enviro-economic elements of sustainable development involving two commodities. Singh et al.^30^ examined the environmental factors related to ginger drying in a hybrid active greenhouse sustainable development.

Fixed solar systems exhibit several limitations, as their efficiency is contingent upon the amount of solar radiation they receive. Consequently, the pursuit of innovative methods to enhance the thermal efficiency of solar collectors is essential within this sector. Numerous initiatives are underway to improve the thermal efficiency (TE) of solar collectors, using different ways such as, an automatic solar collector tracker^31–34^, solar reflectors compound parabolic concentrator^35,36^, a heat pipe evacuated tube^36,37^, a circular glass tube^38^, a heat pipe^39^, an individual spiral-shaped SC tube^40^, Scheffler solar concentrator^41^, and a mirror-reflected^42^.

Hence, the current study aims to develop and assess the performance of a new solar dryer with TSFPSC to enhance the use of solar energy for drying tilapia fish fillets of different slice thicknesses. The study aims to compare the data acquired from this procedure with that of open-air drying utilizing multiple criteria. The testing procedure will entail drying tilapia fish fillets at different slice thicknesses to ascertain the best thickness for drying efficiency. The data acquired from the solar dryer will be juxtaposed with data gathered from open-air drying utilizing several parameters. This study aims to enhance comprehension of solar energy application in the drying of tilapia fish fillets and to aid in the advancement of sustainable, cost-efficient drying methods. Furthermore, the second aim of the current paper was undertaken to discern the techno–enviro–economic performances by utilizing a DSD to optimize the utilization of solar energy for drying STFF of varying slice thickness. To determine the EP time, and net CO_2_ mitigation over the lifetime of the DSD. Through this study, we hope to add to the body of knowledge already available on the drying kinetics of STFF and shed light on whether using DSD for this purpose would be environmentally feasible.

## Materials and methods

### Materials and experimental set-up

**Fig. 1:**
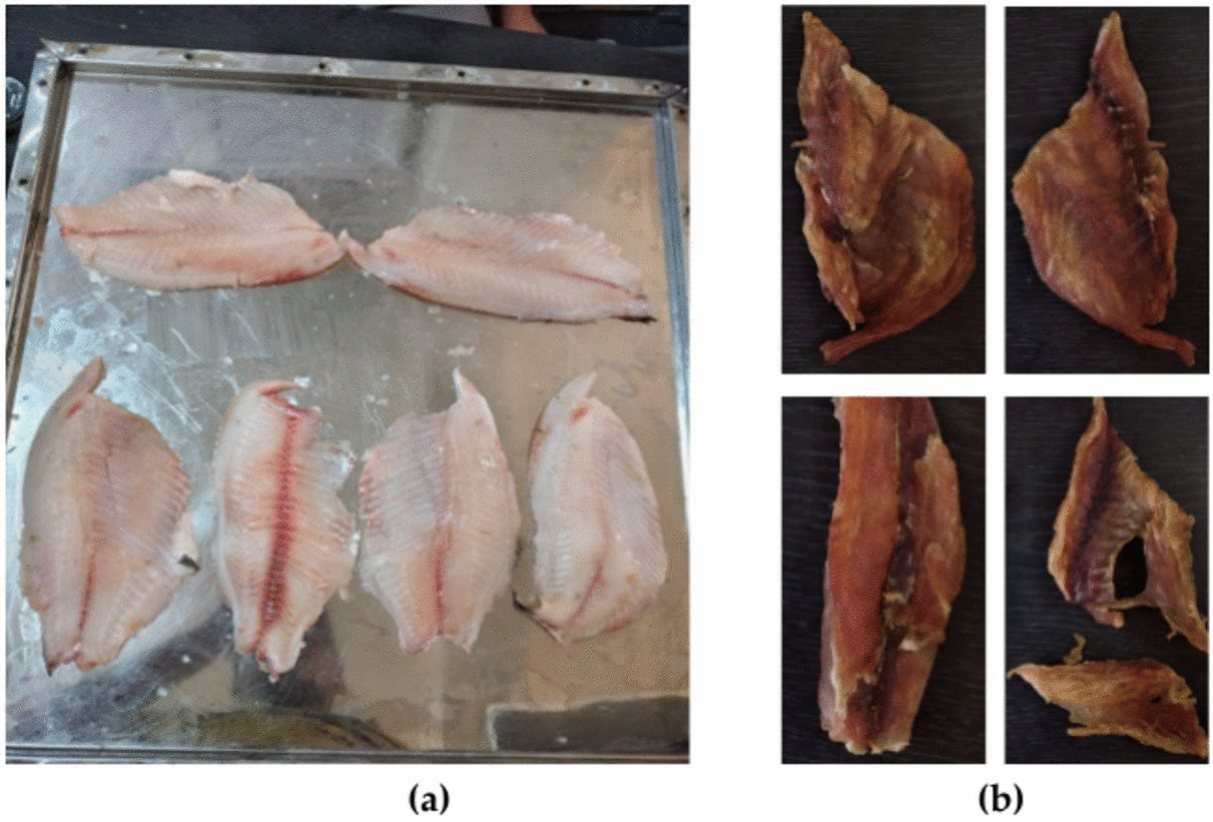
Fresh and dried STFF. Whereas (**a**) is the fresh STFF, and (**b**) is the dried STFF.

All drying tests were conducted at the Agricultural Research Center located in Giza, Egypt, in 2023. The drying process was carried out in the months of June, July, and August 2023, where the average monthly solar insolation was 700, 720, and 750 W/m^2^, and ambient temperature was 33, 35, and 35 °C, respectively. The STFF were dried using the DSD at a constant air speed of 0.5 m/s. Drying tests were carried out from 8 a.m. to 5 p.m. every day. The STFF used for this study was sourced from Lake Nasser in Aswan, Egypt. The effect of fillet thicknesses (4, 8, and 12 mm) was investigated using the open sun drying (OSD) and the developed solar dryer (DSD) at a fixed air speed of 0.5 m/s. Where the fresh fish were washed, cleaned and sliced to required fillet thicknesses of 4, 8, and 12 mm with the recommended fillet thickness that recommended in Ref.^43^, then the fillets were soaked in 5% saline solution for 15 min at room temperature. Figure 1 shows fresh and dried STFF. A STFF with thicknesses of 4, 8, and 12 mm and an initial weight of 100 gm for each slice were used for the drying process. Hourly records of the MC of the STFF samples were taken during the drying process until the equilibrium MC was reached.

### Methods

#### Description of the DSD

**Fig. 2:**
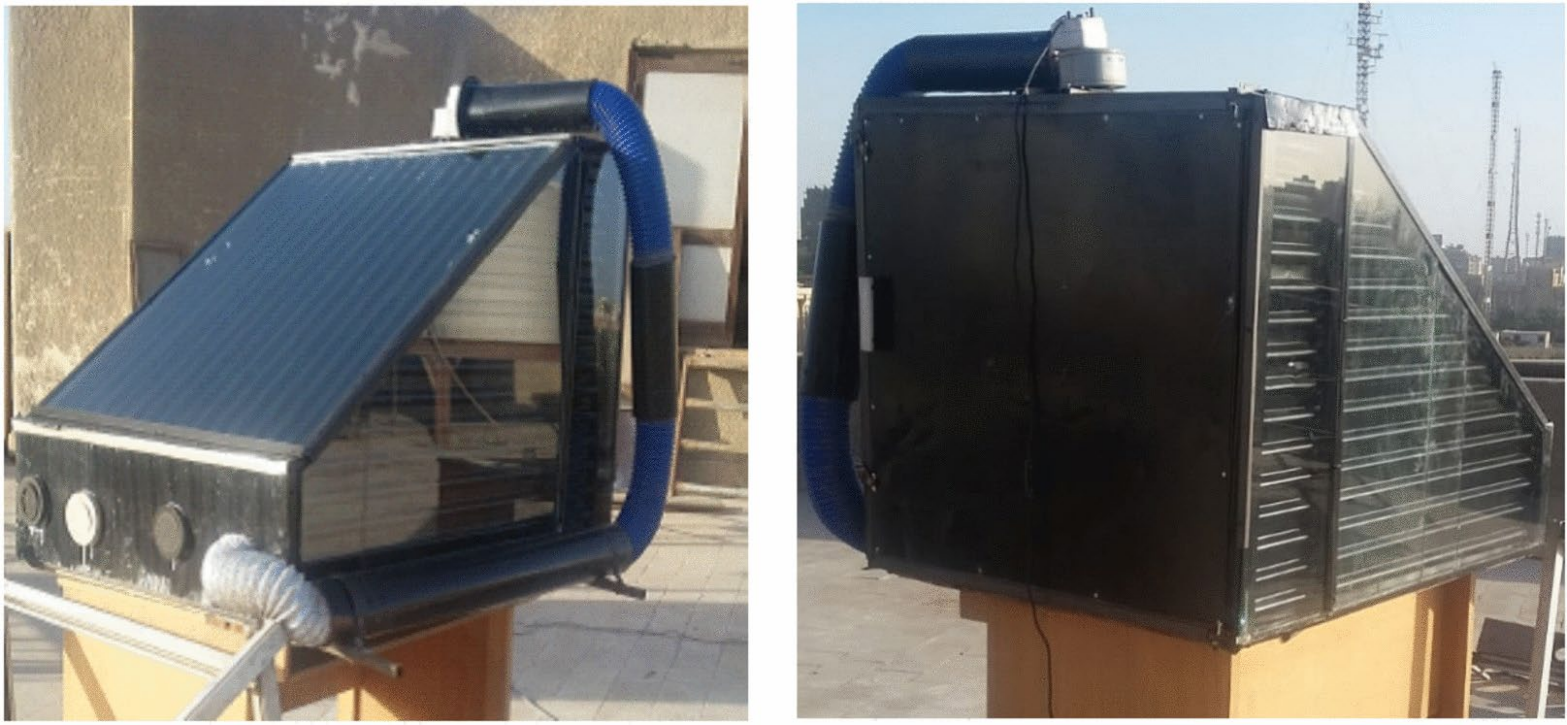
The DSD integrated with a TSFPSC.

Figure 2 shows different views of the DSD integrated with TSFPSC, where it was designed with a 6 kg load capacity of STFF. The DSD has dimensions of 1.00, 0.96, and 0.95 m in length, width, and height, respectively. The drying room features a triangular prism shape and is equipped with TSFPSC on its upper, east, and west sides. The TSFPSC, with a total surface area of 2.434 m^2^, is double insulated walled, featuring a 3 mm thick glass cover that is transparent. The drying room’s back side is a double-wall insulated door with a total volume of 877.34 L, while its four drying trays are made of stainless steel, specifically assigned to carry STFF for drying purposes. The hot air inside the drying cabin is distributed using an electric fan with a power rating of 100 W and a constant air speed of 0.5 m/s. The solar collector was southward direction oriented with an incline angle of 30°. The initial MC of the STFF was 74.83% (w.b.).

#### Evaluation of the DSD

##### Drying parameters

*Moisture content (MC)*

The MC was estimated by heating an STFF sample at 105 ± 1 °C in a hot-air electric oven for 10 h, based on the described method by AOAC^44^. Then, both the initial and final MC of the STFF samples were estimated using Eqs. (1) and (2), as mentioned by^45–48^.

1 $${\mu }_{w}=\left[\frac{{W}_{w}- {W}_{d}}{{W}_{w}}\right]\times 100$$

2 $${\mu }_{d} =\left[\frac{{W}_{w}- {W}_{d}}{{W}_{d}}\right]\times 100$$

where, ${\mu }_{w}$ are the MC on wet basis and dry basis, respectively in %, ${W}_{w}$ is the wet weight, ${W}_{d}$ is dry weight.

*Moisture rate (MR)*

The MR of dried STFF samples at time (t) were estimated using Eq. (3), according to^33,34,48–50^,

3 $$MR=\frac{{M}_{t}-{M}_{e}}{{M}_{0}-{M}_{e}}$$

where ${M}_{t}$, ${M}_{0}$ and ${M}_{e}$ are MC at any time of drying, initial MC and equilibrium MC, respectively in (kg_water_/kg_dry matter_). Using appropriate mathematical models, the moisture ratio was utilized to investigate the kinetics of date fruit drying. The value of ${M}_{e}$ can be disregarded, because it is comparatively minor to the values of ${M}_{t}$ and ${M}_{0}$, Thus, according to Doymaz et al.^51^,the moisture ratio of dates may be written as illustrated in Eq. (4),

4 $$MR=\frac{M}{{M}_{0}}$$

*Drying constant (k)*

An exponential relationship was seen between the drying time and MR, resulting in the determination of the drying constant. The drying constant is crucial for fully characterizing the kinetics of the drying process. And it was determined by using Eqs. (5) and (6), as described in references^52,53^.

5 $$\frac{dM}{{dt}} = - k \times \left( {M - M_{e} } \right)$$

where Eq. (5) was obtained by integrating Eq. (4) as mentioned by^54–56^,

6 $$MR=A\text{exp}(-k\times t)$$

*Effective moisture diffusivity (EMD)*

During the decreasing rate stage, Fick’s diffusion formula could be utilized to describe the drying process of biological products. (Eq. (7))^57^.

7 $$\frac{\partial M}{{\partial t}} = D_{eff} \left( {\frac{{\partial^{2} M}}{{\partial r^{2} }} + \frac{2}{r}\frac{\partial M}{{\partial r}}} \right)$$

With the appropriate initial and boundary conditions:

8 $${M\left.\left(r,t\right)\right|}_{t=0}= {M}_{0}$$

9 $${\left.\frac{\partial M\left(r,t\right)}{\partial M}\right|}_{t=0}= 0$$

10 $${M\left.\left(R,t\right)\right|}_{t>0}= {M}_{e}$$

The variable *M* represents the MC, w.b. The variable *t* represents the drying time, measured in seconds. Where, Eq. (10) may be expressed as Eq. (11).

11 $${\text{M}\left.\left(\text{R},\text{t}\right)\right|}_{\text{t}>0}= 0$$

By using the numerical approach, let us assume a solution to the above form in order to separate the variables,

12 $$M\left( {r,t} \right) = F\left( r \right) * G\left( t \right)$$

where F is function of $\left(r\right)$ only, and G is function of $\left(t\right)$ only. Combining Eqs. (7) and (12) using the initial and boundary conditions,

13 $$M\left( {r,t} \right) = M_{0} \left( {1 + \frac{2R}{\pi }\mathop \sum \limits_{n = 0}^{\infty } \left( {\frac{{\left( { - 1} \right)^{n + 1} }}{n}\frac{1}{r}\sin \left( {\frac{n \times \pi \times r}{R}} \right)\exp \left( { - \frac{{D_{eff} \times n^{2} \times \pi^{2} \times t}}{{R^{2} }}} \right)} \right)} \right)$$

The rate of transfer at time $\left(t\right)$ across the surface of the sphere is,

14 $$4\pi {R}^{2}{N}_{A}\left(t\right)= -4\pi \times {R}^{2}{\times D}_{eff}$$

with evaluating $\frac{\partial M}{{\partial r}}$ at r = R form Eq. (14),

15 $$4\pi {R}^{2}{N}_{A}\left(t\right)=8\pi {\times R}^{2}\times {M}_{0}\times {D}_{eff}\sum_{n=1}^{\infty }\left(exp\left(-\frac{{D}_{eff\times }{n}^{2}{\times \pi }^{2}\times t}{{R}^{2}}\right)\right)$$

The total transfer per unit surface up to time t, *N*_*A*_ is, where,

16 $$\frac{{N}_{A}^{-}}{4\pi {R}^{2}}={\int }_{0}^{t}{N}_{A}\left(t\right)dt= {M}_{0}\frac{R}{3}\left(1-\frac{6}{{\pi }^{2}}\sum_{n=0}^{\infty }\left(\frac{1}{{n}^{2}}exp\left(-\frac{{D}_{eff}{\times n}^{2}{\times \pi }^{2}\times t}{{R}^{2}}\right)\right)\right)$$

A material balance on the transfer up to time $\left(t\right)$ is,

17 $$\frac{4\pi \times {R}^{3}}{3}\left({M}_{0}-M\right)= {N}_{A}^{-}$$

where M is the average MC throughout the sphere at time t. combining Eqs. (18) and (19),

18 $$\frac{{M}_{0}-M}{{M}_{0}}=1-\frac{6}{{\pi }^{2}}\sum_{n=0}^{\infty }\left(\frac{1}{{n}^{2}}exp\left(-\frac{{D}_{eff}\times {n}^{2}\times {\pi }^{2}\times t}{{R}^{2}}\right)\right)$$

By simplify Eq. (18),

19 $$\frac{M}{{M}_{0}}=\frac{6}{{\pi }^{2}}\sum_{n=0}^{\infty }\left(\frac{1}{{n}^{2}}exp\left(-\frac{{D}_{eff}{\times n}^{2}{\times \pi }^{2}\times t}{{R}^{2}}\right)\right)$$

As the MR previous Equation and form third condition; the Eq. (20) was simplified,

20 $$MR= \frac{M}{{M}_{0}}=\frac{6}{{\pi }^{2}}\sum_{n=0}^{\infty }\left(\frac{1}{{n}^{2}}exp\left(-\frac{{D}_{eff}{\times n}^{2}\times {\pi }^{2}\times t}{{R}^{2}}\right)\right)$$

The determination of diffusion coefficients is often achieved by graphing experimental drying data as ln (MR) vs drying time (t). This plot yields a straight line with a slope equal to ${\pi }^{2}\frac{{D}_{eff}}{{R}^{2}}$, as reported by^58–62^,

21 $$\text{ln}\left(MR\right)= \text{ln}\frac{6}{{\pi }^{2}}- \left(\frac{{{\pi }^{2}\times D}_{eff}}{{R}^{2}}\right)t$$

Thin-layer drying models (TLDM).

Table 1 displays a variety of TLDM used for the analysis and description of experimental data collected during the drying process of STFF. The non-linear regression analysis was used to compute the optimal values of the model parameters in Eqs. (22), (23), (24) Microsoft Excel 2016 (KB4011684) 64-Bit Edition (https://www.microsoft.com/en-us/download/details.aspx?id=56547). The suitability of the equations for fitting the experimental data was evaluated and compared using models contestants, the determination coefficient (R^2^), the chi-square (χ^2^), and the root mean square error, as described by Rabha et al.,^49^.

**Table 1:** Selected TLDM to demonstrate the drying process of STFF.

No	TLDM name	Equation*	Ref
1	Newton (Lewis)	$MR=exp\left(-kt\right)$	^66^
2	Page	$MR=exp\left(-k{t}^{n}\right)$	^57,67^
3	Combined two-term and page	$MR=a exp\left(-k{t}^{n}\right)+b exp\left(-h{t}^{n}\right)$	^68^
4	Modified Henderson and Pabis	$MR=a\text{exp}\left(-kt\right)+b exp\left(-gt\right)+c exp\left(-ht\right)$	^69^
5	Modified Midilli II	$MR=a exp\left(-k{t}^{n}\right)+b$	^22^
6	Modified Page III	$MR=k \text{exp}{\left(-\frac{t}{{d}^{2}}\right)}^{n}$	^21^
7	Modified Two Term III	$MR=a \text{exp}\left(-kt\right)+\left(1-a\right) exp\left(-kat\right)$	^22^
8	Logistics	$MR= \frac{b}{1+a\text{exp}\left(kt\right)}$	^70^
9	Simplified Ficks’ diffusion	$MR=a\text{exp}\left(-c\left(\frac{t}{{L}^{2}}\right)\right)$	^58^
10	Approximation of diffusion	$MR=a\text{exp}\left(-kt\right)+\left(1-a\right)exp\left(-kbt\right)$	^71,72^
11	Parabolic model	$MR=a+bt+c{t}^{2}$	^21^

*t is the drying time, h; *L* is the thickness of the samples (slab), m; *a, b, c, d, g, h*, and *n* are the models constants, dimensionless.

22 $${R}^{2}=1-\frac{\sum_{i=1}^{N}{{(MR}_{pre, i}-{MR}_{obs, i})}^{2}}{\sum_{i=1}^{N}{{(\overline{M}R }_{pre}-{MR}_{obs, i})}^{2}}$$

23 $${\chi }^{2}=\frac{\sum_{i=1}^{N}{{(MR}_{pre, i}-{MR}_{obs, i})}^{2}}{N-n}$$

24 $$RMSE=\sqrt{\frac{1}{N}{\sum }_{i=1}^{N}{{(MR}_{pre, i}-{MR}_{obs, i})}^{2}}$$

The experimental and anticipated values for the *i*^*th*^ observation are denoted as ${MR}_{obs, i}$ and ${MR}_{pre, i}$ respectively, whereas ${\overline{M}R }_{pre}$ _pre denotes the average predicted value. N represents the total number of observations, whereas n represents the number of constants in a model.

The TLDM that most closely corresponds to the experimental data was determined using the criteria mentioned earlier^63–65^, selecting the one with the lowest χ^2^ and RMSE values, and the greatest R^2^ value.

*Economic analysis*

An economic analysis helps assess if the DSD is financially viable for you. It considers the initial investment cost, operational expenses, and potential savings on conventional drying (like fuel costs). This helps you determine if the DSD will pay for itself over time^73^. Where the estimation of the economic performance criteria of the DSD was conducted based on the Egyptian financial climate. Where the annualized investment cost (${C}_{a}$) and yearly capital cost (${C}_{ac}$) of the DSD were calculated using Eqs. (25) and (26) as stated by^74–82^.

25 $${C}_{a}={C}_{ac}+{C}_{m}-{V}_{a}$$

where ${V}_{a}$ is the salvage value, and ${C}_{m}$ is maintenance cost where (${C}_{m}=3\% of {C}_{a} \& {V}_{a}=8\% of {C}_{a}$),

26 $${C}_{ac}={C}_{cc}\times {F}_{c}$$

27 $${F}_{c}= \frac{{d(1+d)}^{n}}{{(1+d)}^{n}-1}$$

where n is the lifetime of the DSD (equal to 15 years), ${C}_{cc}$ is total capital cost, ${F}_{c}$ is the capital recovery factor, and *d* is the interest rate (equal to 20%).

The drying cost per kg (${C}_{s}$) and the dried quantity per year (${M}_{y}$) of STFF using DSD were estimated using Eqs. (28) and (29) as reported by^74–76,79,83–85^,

28 $${C}_{s}=\frac{{C}_{a}}{{M}_{y}}$$

29 $${M}_{y}=\frac{{M}_{d}\times D}{{D}_{d}}$$

where, *D* is the drying days per year, ${M}_{d}$ is the amount of STFF every batch, and ${D}_{d}$ is the drying time per batch.

The price of one kg of the dried (${C}_{ds}$) and fresh (${C}_{dp}$) STFF was calculated using Eqs. (30) and (31), as stated by^74–76^,

30 $${C}_{ds}={C}_{dp}+{C}_{s}$$

31 $${C}_{dp}={C}_{fd}\times \frac{{M}_{f}}{{M}_{d}}$$

where, ${M}_{f}$ is the quantity of fresh TFF and ${C}_{fd}$ is the fresh TFF cost.

The savings from DSD following *(j)* years are obtained by Eq. (32),

32 $${S}_{j}=[{SP}_{c}-{C}_{ds}]\times {M}_{d}\times {\left(1+j\right)}^{j-1}$$

where, ${SP}_{c}$ is the selling price of dried STFF per kg.

The payback time *(N)* for DSD was estimated using Eq. (33) as stated by^74–76,86^,

33 $$N=\frac{ln\left[1-\frac{{C}_{cc}}{{S}_{1}}(d-i)\right]}{\text{ln}\left(\frac{1+i}{1+d}\right)}$$

where, *i* represents the inflation rate (equal to 39.7%) and ${S}_{1}$ is the DSD savings after the very first year.

##### Environmental analysis

*Specific energy consumed (SEC)*

The SEC for drying the STFF is calculated using Eqs. (34) and (35) according to^87–89^,

34 $$SEC= \frac{{E}_{in.c}}{{M}_{out}}$$

35 $${E}_{in.c}= {A}_{c}{\int }_{0}^{t}{Ins}_{c}(t)dt$$

where, ${E}_{in.c}$ is the input energy to solar collector, and ${M}_{out}$ is the moisture removed (Mr) from the STFF; ${A}_{c}$ is the solar collector’s surface area in m^2^, ${Ins}_{c}$ is the solar intensity in W/m^2^.

*Embodied energy (EE)*

Table 2 illustrates the EE of materials used in manufacturing of the DSD. Where the EE can be defined as the amount of energy necessary to manufacturing the DSD.

**Table 2:** EE calculation data for manufacturing of the DSD^90–92^.

No	Materials	Specific EE, (kW.h/kg)	Weight, kg	EE (kW.h)
1	Metal frame	55.28	15.0	829.2
2	Glass cover	7.28	15.0	109.2
3	Wood dust	2.0	2.0	4.0
4	Paint	25.11	0.5	12.555
5	Absorber plate	9.636	5.0	48.18
6	Hinges	55.28	0.05	2.764
7	Handel	55.28	0.05	2.764
8	Suction fan			
	1. Metal parts	55.28	0.30	16.584
	2. Motor and cooper wires	19.61	0.20	3.922
9	Drying trays			
	1. Wire mesh	9.67	2.0	19.34
	2. Metal frame	55.28	5.0	276.4
Total EE for manufacturing of the DSD (kWh)				1324.91

*Annual thermal outputs (ATO)*

The annual thermal output (${E}_{th,y}$) of the DSD was calculated using Eq. (36), as stated by Eltawil et al.^93^.

36 $${E}_{th,y}=\frac{{M}_{ev.}\times {h}_{L}}{3.6\times {10}^{6}}\times {N}_{dy}$$

where, ${h}_{L}$ is the latent for evaporation in J/kg, and ${M}_{ev.}$ is the amount of moisture evaporated in kg.

*Time of energy payback (* ${\varvec{E}}{\varvec{P}}$ *)*

The time of EP refers to the duration required to payback the EE of the DSD. It is calculated using Eq. (37), as shown by Prakash et al.^94^.

37 $$EP= \frac{EE}{{E}_{th,y}}$$

*CO*_*2*_ *emission*

The emission of CO_2_/year (${E}_{CO2}$) can be determined using Eq. (38), as described by^90^.

38 $${E}_{CO2}= \frac{EE \times 0.98}{n}$$

*Carbon mitigation*

Understanding the annual carbon dioxide (CO_2_) emissions associated with the DSD is essential for assessing its impact on the environment. The assessment of the potential for climate change is determined by the process of reducing CO_2_ emissions. When a client consumes a unit of energy and experiences losses from poor residential equipment, denoted as *La* (assumed to be 10%), the quantity of power actually provided is equal to $\frac{1}{1-{L}_{a}}$ units. The power plant creates energy by multiplying the units of $\frac{1}{1-{L}_{a}}\times \frac{1}{1-{L}_{td}}$, where *L*_*a*_ represents the distribution losses and *L*_*td*_ represents the transmission losses, assuming both losses are 45%. The usage of coal for power generation in the drying process results in an average CO_2_ equivalent intensity of 0.98 kg/kWh^20^.

39 $$The {CO}_{2} mitigation per kWh of the DSD = \frac{1}{1-{L}_{a}}\times \frac{1}{1-{L}_{td}} \times 0.98= 1.9798$$

As stated by Nayak et al.^95^, the carbon dioxide (CO_2_) reduction throughout the whole lifespan of the DSD is:

40 $$The {CO}_{2} mitigation over the system lifetime \left(kg\right)= EE\times n\times 1.9798$$

The net decrease in CO_2_ emissions throughout the lifespan of the DSD was calculated by subtracting the total CO_2_ emissions mitigation potential from the total CO_2_ emissions contained in the DSD.

41 $$Net \, mitigation \, over \, the \, lifetime \, \left( {kg} \right) \, = \, Total \, CO_{2} mitigation - Total \, CO_{2} emission$$

42 $$\text{Net mitigation over the lifetime }(\text{kg}) =\left({E}_{th,y}\times n\right)\times 1.9798 -EE$$

## Results and discussion

### Drying parameters

#### Moisture removal (Mr) from different STFF samples

**Fig. 3:**
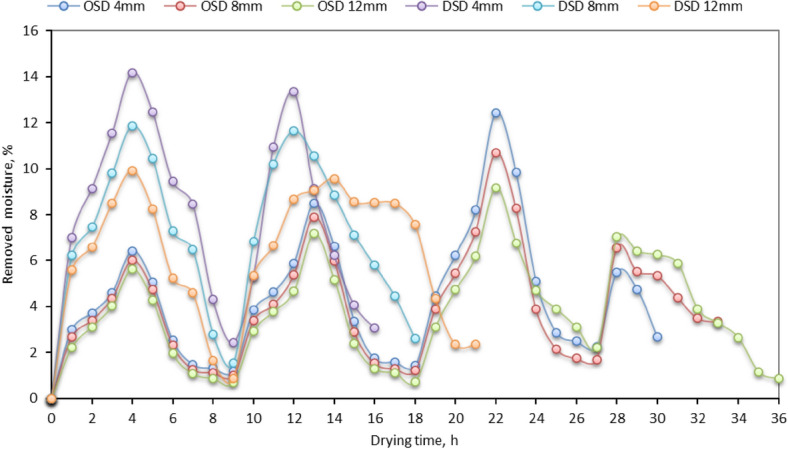
Removed moisture of different STFF samples for the OSD and the DSD at different thicknesses.

Figure 3 shows the amount of Mr from various STFF samples for both drying systems (OSD and the DSD) at different fillet thicknesses. Where it was observed that Mr is different for different drying systems and different fillet thicknesses. We found that for 4 mm fillet thickness (DSD), the Mr was the highest after one hour from the start of the experiment. It was also observed that for a higher Mr (14%), On comparing, it concluded that the less Mr gets more time for Mr rate. In addition, we observed that the highest amount of moisture was removed on the first day, while the lowest amount was removed on the last day. The Mr decreased as the drying process progressed. It reached the equilibrium state between the internal water vapor pressure inside the drying STFF samples and the partial pressure of the hot air inside the drying room. The obtained results come in agreement with Singh and Gaur^76^ and Khater et al.^96^, who demonstrated that a large amount of moisture evaporated from the dried crop during the first day of the drying process, and this amount decreases with the time consumed. On the other hand, the plotted data in Fig. 3 showed the highest daily Mr happens at mid-day. It has been seen that the rate of Mr increases after 1.0 pm compared to before 1.0 pm. This is because the sun irradiation is greater after 1.0 pm on the best performance days, as shown by Slam et al.^97^.

#### MC for different STFF samples

**Fig. 4:**
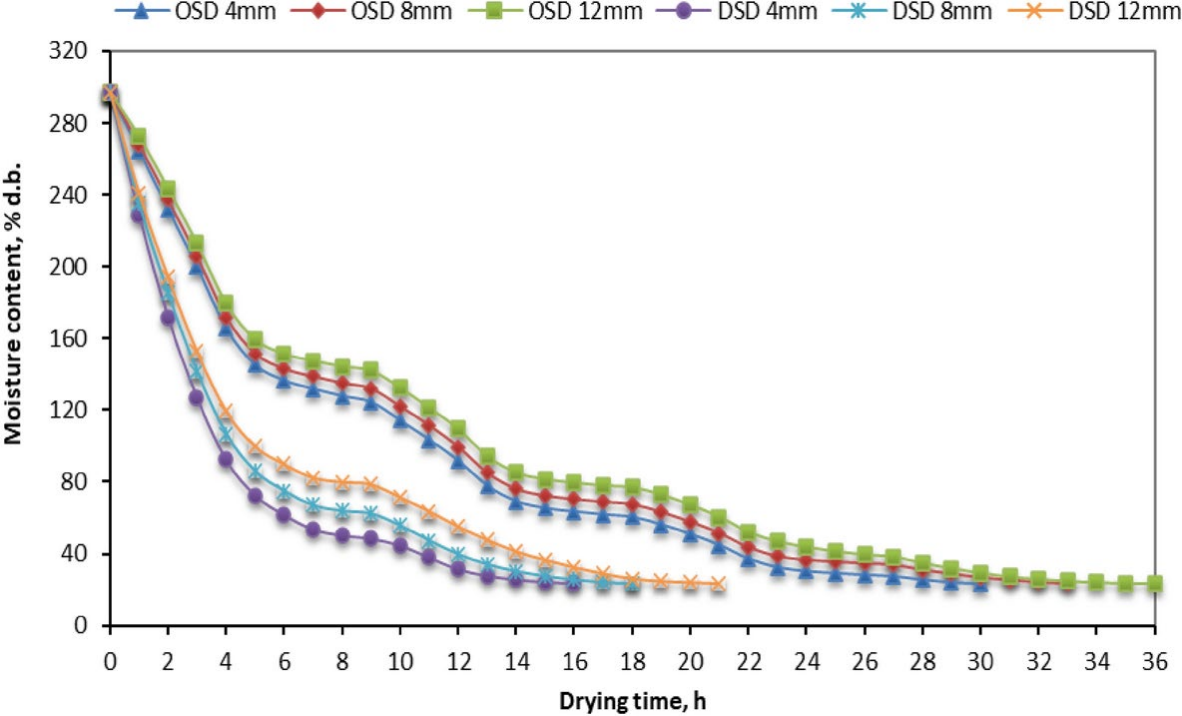
MC of different STFF samples for the OSD and the DSD at different fillet thicknesses.

Figure 4 presented the effect of the examined drying systems (OSD and DSD) and fillet thicknesses (4, 8, and 12 mm) of STFF on MC. Where the higher temperature in the drying room of the DSD leads to a faster DR and a shorter drying time, indicated by the fact that drying times reach the final average MC of 18.84% (d.b.) were 16, 18, 20.5 h, for fillet thicknesses of 4, 8, and 12 mm, respectively. At the same time, the dried STFF in OSD reached the equilibrium MC of 18.84% (d.b.) after 30, 32.5, and 36 h for fillet thicknesses of 4, 8, and 12 mm, respectively. As the air temperature used for drying rises, the rate at which moisture moves from the interior of the STFF to the surface layer also increases. This leads to an increase in the rate of moisture evaporation from the surface layer, resulting in a higher drying rate^98–102^. This relationship is shown in Fig. 4.

#### Moisture rate (MR) for different STFF samples

**Fig. 5:**
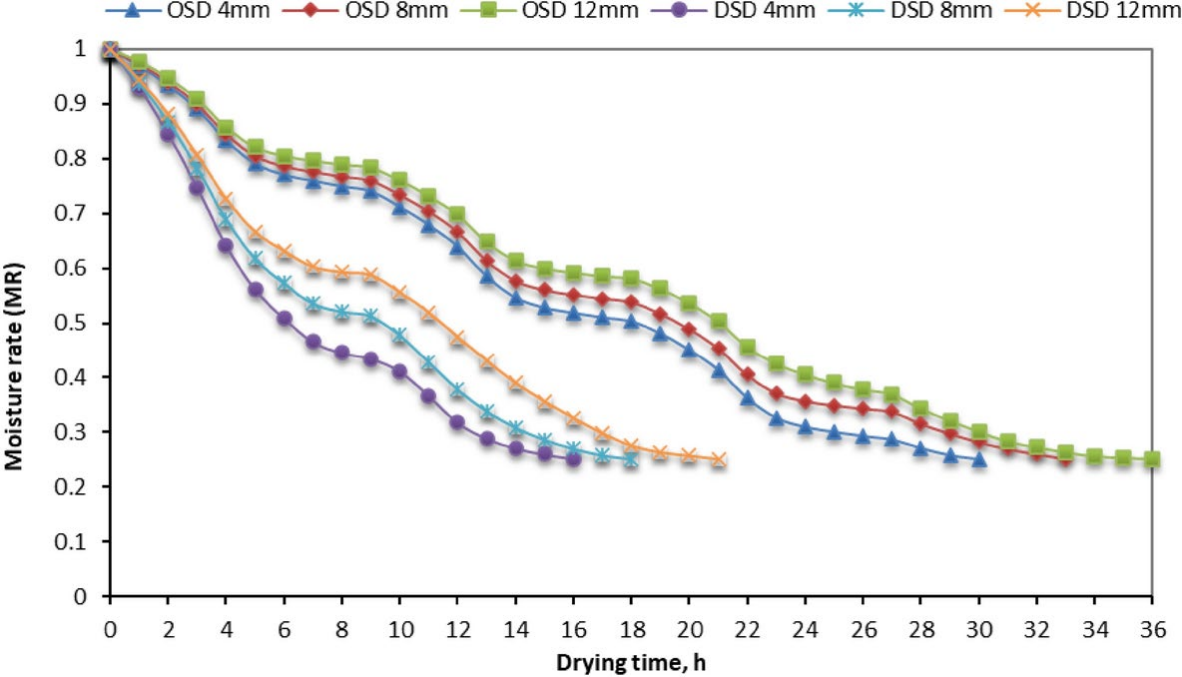
Moisture rate of different STFF samples for the OSD and the DSD at different fillet thicknesses.

Derived from the mean weight of several samples of STFF. Where the relation between MR and drying time was plotted in Fig. 5. From data illustrated in Fig. 5, we found that the STFF samples dried in the OSD had a higher MR than the other STFF samples dried on the DSD. However, the drying time of dried STFF on the DSD was reduced by about 53.3%, 54.55%, and 61.11% for fillet thicknesses of 4, 8, and 12 mm compared with the other STFF samples dried in DSD. The drying process was expedited by the lowest MR, facilitating the removal of moisture and shortening the duration of drying^98–100^. In addition, we found that MR curves showed a significant fast reduction in the MR during the first stage of the drying process, which comes in agreement with Ambawat et al.^101^.

### Experiment drying constant (k) and coefficient of determination (R^2^)

Table 3 presents the experiment drying coefficient (k) and coefficient of determination (R^2^) for several STFF samples and the drying systems they were subjected to. When the drying air temperature inside the DSD increases, the drying coefficient (k) also increases in comparison to the OSD. The findings were in line with the same pattern seen in the drying rate data. which aligns with the studies of Doymaz^103^; Kaleta et al.,^104^; Meziane,^105^, and Metwally et al.^20^ who made similar conclusions. However, there is an inverse relationship between the drying coefficient and fillet thickness of the STFF samples, where the calculated value of the drying coefficient decreased from 0.048 to 0.041 while the fillet thickness of the STFF samples increased, as illustrated in Table 3. This effect may be due to decreasing the internal temperature of the STFF samples while increasing the fillet thickness.

**Table 3:** Experiment drying coefficient and determination coefficient of STFF drying experiment using OSD and DSD.

Thickness, mm	OSD		DSD	
	k	R^2^	k	R^2^
4	0.048	0.9813	0.089	0.9833
8	0.043	0.9872	0.079	0.99
12	0.041	0.9829	0.068	0.9871

### Effective moisture diffusivity (EMD)

**Fig. 6:**
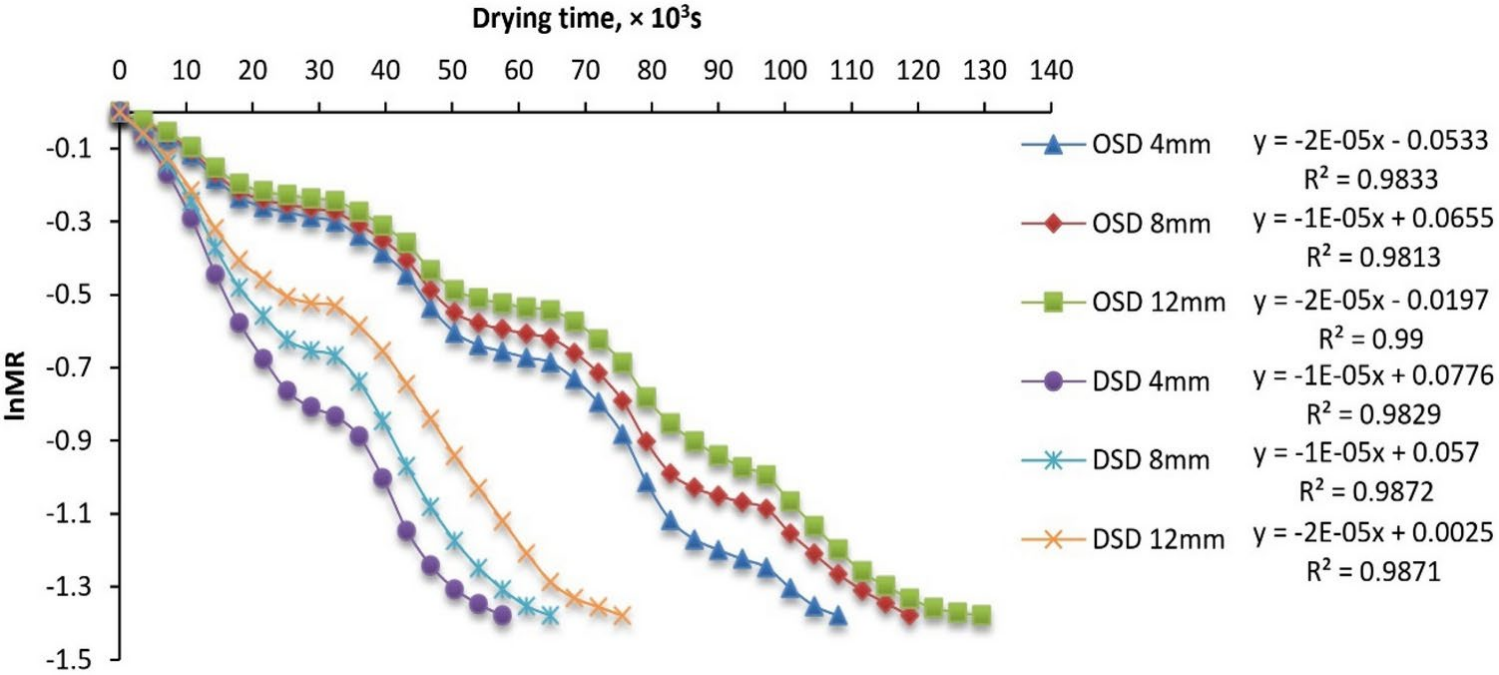
lnMR vs drying time for different drying systems and fillet thicknesses.

Experiment data of MC has converged to draw portraits of ln (MR) along time *(t)* (Fig. 6). A linear association between drying time and ln (MR) was seen in both drying methods. The primary experimental parameter often used for simulating drying processes is the reduction in sample mass, which is quantified as the ratio between the water content at a certain time (*t*) and the beginning MC^106^.

**Fig. 7:**
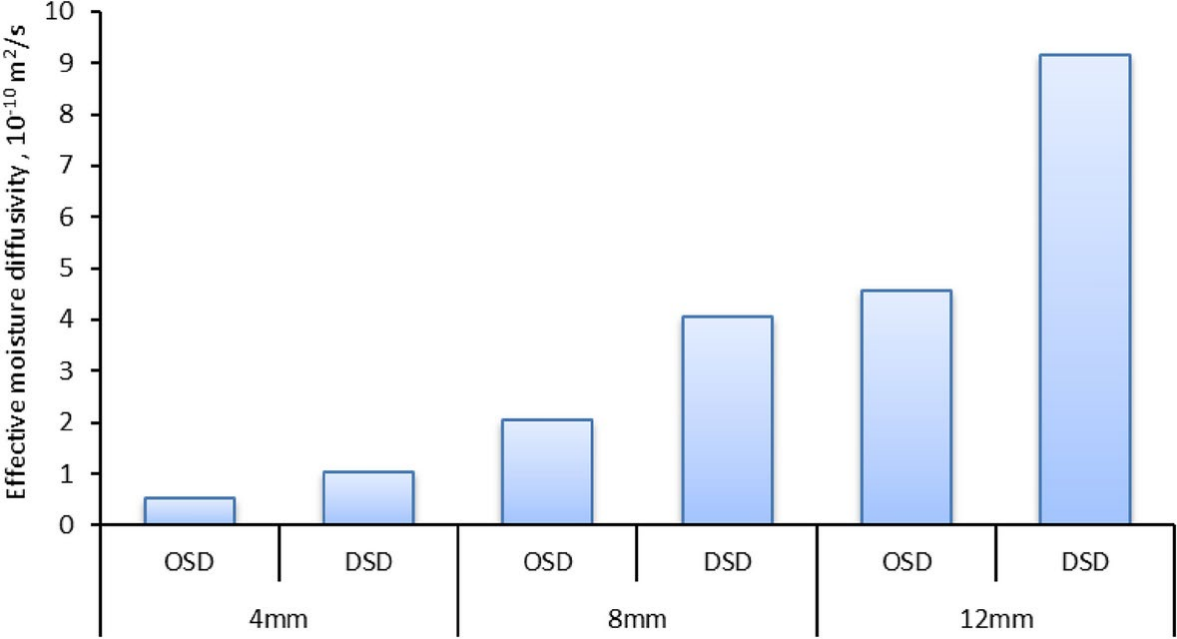
EMD of STFF for different drying systems and fillet thicknesses.

The findings shown in Fig. 7 indicate that the drying time is mostly determined by the internal mass transfer resistance, which is influenced by the existence of a decreasing rate drying phase. Consequently, the EMD values for the drying experiment under various circumstances are determined using Fick’s second law. The EMD of several samples of STFF with thicknesses of 4, 8, and 12 mm was determined for both drying methods. The EMD values ranged from 0.51 × 10^−10^ to 9.16 × 10^–10^ m^2^/s, which aligns with the findings of prior investigations as shown in Table 4. Furthermore, according to the data shown in Fig. 7, the dried STFF samples on DSD exhibited the greatest values of the EMD when compared to the other STFF samples with the same fillet thickness dried in OSD. It may be elucidated in the following manner: The rise in air temperature will enhance the movement of water molecules, leading to an increase in the rate of water diffusion. This, in turn, improves the heat and mass exchange between the solid–liquid film and hot air. As a result, the MC and partial pressure of water vapor on the surface of the fillet decrease, thereby accelerating the internal evaporation, migration, and EMD process within the STFF^107^.

**Table 4:** Previous studies of the EMD of dried product.

Reference	Dried product	EMD, m^2^/s
Ambawat et al.^101^	Moringa oleifera leaves	3.59–2.92 × 10^−10^
Guan et al.^107^	TFF	6.55–1.23 × 10^−9^
Sandeepa & Rao^108^	Sorghum seeds	3.01–5.50 × 10^−10^
Akpinar & Bicer^64^	Strawberry	4.52–9.63 × 10^−10^
Doymaz^109^	Apricot	6.76–12.6 × 10^−10^
Aghbashlo & Samimi-Akhijahani^110^	Berberis	3.32–90 × 10^−10^
Elfar et al.^111^	TFF	3.47–6.86 × 10^−10^
Ruiz-Cabrera et al.^112^	Cactus pears	1.51–5.32 × 10^−10^
Metwally et al.^20^	Date fruit	0.714–2.17 × 10^−11^
Current study	STFF	0.51–9.16 × 10^–10^

### Thin-layer modeling

The use of mathematical models for thin-layer modeling is essential in determining the drying kinetics of dried products. In order to achieve efficient process design, energy integration, optimization, and control, it’s crucial to possess an effective model^20^. Table 5 displays the eleven chosen fundamental TLDM that were used to characterize the drying kinetics of STFF in both the OSD and DSD drying systems employed in the present investigation.

**Table 5:** TLDM constants and statistical analysis results of (a) OSD and (b) DSD.

Thickness, mm	Model name	Constants									Statistical measures		
		k	a	b	c	d	g	h	L	n	R^2^	${\chi }^{2}$	RMSE
(a) OSD													
4	Newton (Lewis)	0.04251									0.980844	0.001075	0.032235
	Page	0.02684								1.15935	0.988842	0.000648	0.024602
	Combined Two-Term and Page	0.01310	0.93171	0.08006				0.82854		1.36652	0.991750	0.000537	0.021155
	Modified Henderson and Pabis	0.04431	0.34284	0.34284	0.34284		0.04431	0.04431			0.983293	0.001088	0.030104
	Modified Midilli II	0.02044	0.97300	0.00000						1.23834	0.989893	0.000658	0.023414
	Modified Page III	1.02852				2.23341				0.22104	0.983293	0.001088	0.030104
	Modified Two Term III	0.042513	1.000002								0.980844	0.001247	0.032235
	Logistics	0.07148	0.70596	1.67424							0.991182	0.000574	0.021870
	Simplified Ficks Diffusion		1.02852		0.22104				2.23341		0.983293	0.001088	0.030104
	Approximation diffusion or (Diffusion Approach)	0.04251	1.00000	1.00000							0.980844	0.001247	0.032235
	Parabolic model		0.99072	− 0.03366	0.00028						0.991430	0.000558	0.021560
8	Newton (Lewis)	0.03877									0.984525	0.000847	0.028660
	Page	0.02541								1.14182	0.991103	0.000503	0.021731
	Combined Two-Term and Page	0.01612	0.95303	0.05256				0.82802		1.26685	0.993008	0.000437	0.019265
	Modified Henderson and Pabis	0.04029	0.34216	0.34216	0.34216		0.04029	0.04029			0.986652	0.000835	0.026618
	Modified Midilli II	0.02000	0.97590	0.00000						1.20909	0.991879	0.000508	0.020763
	Modified Page III	1.02648				2.28427				0.21025	0.986652	0.000835	0.026618
	Modified Two Term III	0.038767	1.000002								0.984525	0.000968	0.028660
	Logistics	0.06160	0.85841	1.83081							0.992768	0.000452	0.019592
	Simplified Ficks Diffusion		1.02648		0.21025				2.28427		0.986652	0.000835	0.026618
	Approximation diffusion or (Diffusion Approach)	0.03877	1.00000	1.00000							0.984525	0.000968	0.028660
	Parabolic model		0.99301	− 0.03155	0.00026						0.992796	0.000451	0.019554
12	Newton (Lewis)	0.03553									0.978888	0.001190	0.034016
	Page	0.01955								1.19471	0.990350	0.000560	0.022998
	Combined Two-Term and Page	0.00925	0.93486	0.07824				0.82734		1.40138	0.993597	0.000408	0.018734
	Modified Henderson and Pabis	0.03738	0.34509	0.34509	0.34509		0.03738	0.03738			0.982522	0.001112	0.030951
	Modified Midilli II	0.01361	0.96754	0.00000						1.29518	0.991803	0.000522	0.021196
	Modified Page III	1.03528				2.30071				0.19787	0.982522	0.001112	0.030951
	Modified Two Term III	0.035531	1.000002								0.978888	0.001344	0.034016
	Logistics	0.06384	0.57329	1.54125							0.9931626	0.0004352	0.0193584
	Simplified Ficks diffusion		1.03528		0.19787				2.30071		0.982522	0.001112	0.030951
	Approximation diffusion or (diffusion approach)	0.035531	1.000000	1.000000							0.9788882	0.0013437	0.0340163
	Parabolic model		0.992992	− 0.02779	0.000176						0.993043	0.000443	0.019527
(b) DSD													
4	Newton (Lewis)	0.09696									0.984932	0.000941	0.029698
	Page	0.11771								0.91122	0.988558	0.000765	0.025879
	Combined two-term and page	0.13258	1.02669	0.00000				0.76340		0.87229	0.989551	0.000890	0.024731
	Modified Henderson and Pabis	0.09548	0.32960	0.32960	0.32960		0.09548	0.09548			0.985277	0.001254	0.029356
	Modified Midilli II	0.12654	0.82387	0.19032						1.07826	0.992584	0.000631	0.020835
	Modified page III	0.98881				2.11456				0.42694	0.985277	0.001254	0.029356
	Modified two term III	0.09696	1.000002								0.984932	0.001283	0.029698
	Logistics	0.09553	395.22024	391.15976							0.985226	0.001258	0.029407
	Simplified Ficks diffusion		0.988807		0.426943				2.114556		0.985277	0.001254	0.029356
	Approximation diffusion or (diffusion approach)	0.09696	1.00000	1.00000							0.984932	0.001283	0.029698
	Parabolic model		0.995559	− 0.09253	0.002945						0.988092	0.001014	0.026400
8	Newton (Lewis)	0.08174									0.990331	0.000578	0.023365
	Page	0.09145								0.95171	0.991337	0.000550	0.022116
	Combined two-term and page	0.09900	1.01475	0.00000				0.76484		0.92613	0.991628	0.000655	0.021742
	Modified Henderson and Pabis	0.08099	0.33113	0.33113	0.331134		0.080986	0.080986			0.990457	0.000746	0.023213
	Modified Midilli II	0.09862	0.98451	0.02841						0.94731	0.991642	0.000653	0.021724
	Modified page III	0.99340				1.99614				0.32269	0.990457	0.000746	0.023213
	Modified two term III	0.081736	1.000001								0.990331	0.000756	0.023365
	Logistics	0.08130	427.11039	426.08824							0.990427	0.000748	0.023248
	Simplified Ficks diffusion		0.99340		0.32269				1.99614		0.990457	0.000746	0.023213
	Approximation diffusion or (diffusion approach)	0.08174	1.00000	1.00000							0.990331	0.000756	0.023365
	Parabolic model		0.98758	− 0.07350	0.00182						0.988927	0.000866	0.025004
12	Newton (Lewis)	0.06722									0.987790	0.000692	0.025670
	Page	0.07075								0.97949	0.987962	0.000718	0.025489
	Combined two-term and page	0.06641	0.98798	0.00890				0.77657		0.99799	0.988159	0.000839	0.025280
	Modified Henderson and Pabis	0.06648	0.33074	0.33074	0.330743		0.06648	0.06648			0.987973	0.000852	0.025478
	Modified Midilli II	0.06850	0.99509	0.00000						0.98967	0.987991	0.000851	0.025458
	Modified page III	0.99223				2.05648				0.28115	0.987973	0.000852	0.025478
	Modified two term III	0.06722	1.000002								0.987790	0.000865	0.025670
	Logistics	0.07259	5.89497	6.78279							0.987868	0.000859	0.025589
	Simplified Ficks Diffusion		0.992232		0.281153				2.056481		0.987973	0.000852	0.025478
	Approximation diffusion or (diffusion approach)	0.06722	1.00000	1.00000							0.987790	0.000865	0.025670
	Parabolic model		0.97349	− 0.05557	0.00099						0.986924	0.000926	0.026566

**Fig. 8:**
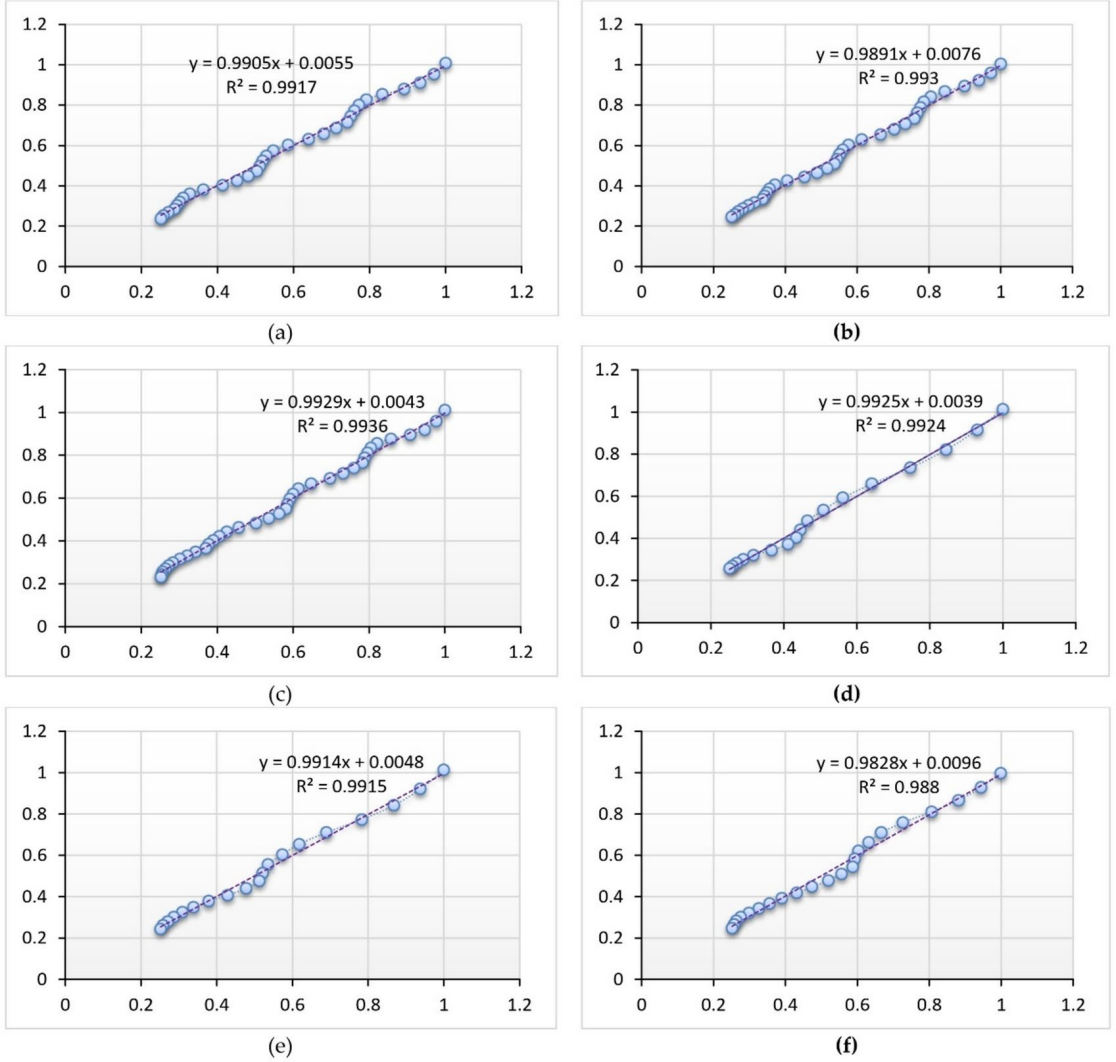
The experimental and predicted MR for drying STFF. Whereas (**a**–**c**) indicate that the combined two-term and page models are the best TLDM for describing the drying behavior of the OSD across all fillet thicknesses. In contrast, (**d**,**e**) demonstrate that the modified Midilli II model is the most effective TLDM for characterizing the drying behavior of the DSD for fillet thicknesses of 4 mm and 8 mm. Finally, (**f**) shows that the combined two-term and page model is also the best TLDM for describing the drying behavior of the DSD at a fillet thickness of 12 mm.

Choosing an appropriate TLDM is essential for precisely forecasting the drying characteristics of different items. After identifying an appropriate model, the next step is to search for secondary models that may help forecast drying curves in non-isothermal situations^20^. Nevertheless, the choice of the most suitable TLDM for characterizing the drying characteristics of vegetables and fruits can’t exclusively rely on the quantity of constants. The selection procedure must be driven by statistical indications that have proven effective in choosing appropriate drying models, as documented in the literature. Hence, it’s crucial to meticulously evaluate these statistical indicators when choosing a TLDM, guaranteeing that the TLDM selection is based on well-informed decisions and supported by actual data. R^2^, reduced χ^2^, and RMSE are statistical indicators used to evaluate the quality of the fitted models. Where previous studies of^20,63–65,113–116^ have shown that the TLDM with the highest R^2^, lowest χ^2^, and RMSE values is the most appropriate for establishing the TLDM of date fruits. The statistical results of various TLDM, are shown in Table 6. In addition, all eleven basic TLDM were applied to predict the drying behavior of STFF during the drying process, while the Combined Two-Term and Page model had the best fitting for the OSD system, and the Modified Midilli II model and Combined Two-Term and Page model had the best fitting for the DSD system (Fig. 8).

**Table 6:** The most appropriate TLDM for describing the drying behavior of STFF for both OSD and DSD at different fillet thicknesses.

Thickness, mm	OSD	DSD
4	Combined two-term and page	Modified Midilli II
8	Combined two-term and page	Modified Midilli II
12	Combined two-term and page	Combined two-term and page

### Economic analysis

The performance study of DSD involves a quantitative evaluation of economic processes that may assist policymakers, investors, and food processors in making informed decisions on crop drying systems. The research used Eqs. (25), (26), (27), (28), (29), (30), (31), (32), (33), in which the lifecycle reduction strategy combines the simple recovery methodology. The analysis took into account significant variables, as seen by the data presented in Tables 7 and 8, while also taking into consideration the state of the Egyptian economy and the anticipated expenses associated with the DSD components. As illustrated in Table 7, the manufacturing and construction cost or capital cost of the DSD is only 105 USD, which is very low compared with the other available drying systems, and the expected life of this DSD is 15 years. According to the data listed in Table 7, the annual capital and investment cost were 22.458 USD and 21.334 USD, respectively.

**Table 7:** Various cost related to the DSD for drying STFF.

Cost parameters	Unit
Capital cost, USD	105
Lifespan, years	15
Annual capital cost, USD	22.458
Annual maintenance cost, USD	0.673
Annual salvage value, USD	1.7966
Annualized investment cost, USD	21.334

**Table 8:** Economic parameters of the DSD for drying STFF.

Economic parameters	Unit
Mass of STFF per batch, kg	6.00
Quantity of dried STFF annually, kg	273.75
Drying cost of per kg of STFF, USD	0.0195
Cost of 1 kg fresh STFF, USD	0.412
Cost of fresh TFF per kg of dried product, USD	1.648
Selling price per kg of STFF, USD	3.0
Saving after 1 year, USD	370.11
Payback time, years	0.356

The data shown in Table 8 demonstrates that the DSD has the capacity to yield an annual production of 273.75 kg of dried STFF. This may result in substantial cost savings, amounting to a total of 370.11 USD per year. Furthermore, the study revealed that the payback period of the DSD was around 0.356 years or less than half a year (less than five months), thus demonstrating its cost-effectiveness.

### Environmental analysis

Table 9 presents the examination of many environmental factors of the DSD. The results showed that the specific energy consumption was 5.77 kWh/kg, and the EE was 1324.91 kW.h. Furthermore, computations were conducted to ascertain the yearly thermal energy generated by the DSD, which amounted to 514.89 kW.h. Additionally, we determined that the EP period was 1.59 years. This pertains to the time it takes for the DSD to regain the energy used during its functioning, as quantified by this measurement. In Addition, the total reduction in CO_2_ emissions for the lifespan of the DSD was 14 tons. These results are inferior to several recent research shown in Table 10.

**Table 9:** Environmental parameters of the DSD.

Environmental parameters	
Specific energy consumed, kW.h/kg	5.77
Embodied energy, kW.h	1324.91
Annual thermal output, kW.h	514.89
EP time, year	1.59
The CO_2_ mitigation over the DSD lifetime, tons	39.3
Net CO_2_ mitigation over the lifetime, tons	14.0

**Table 10:** Comparison between EP time of our DSD with other similar drying systems.

Reference	Type of the SD	Dried product	Energy payback time, years
Brahma et al.^117^	Phase change material SD	Tomato	2.51–2.98
Sharshir et al.^29^	Solar air heaters	Eggplant & Grapes	1.27–8.41
Andharia et al.^118^	A mixed-mode SD	Agricultural products	2.70
Zeeshan et al.^119^	Indirectly forced SD	Tomato	5.1396
Metwally et al.^20^	Automatic SD	Date fruit	7.54–7.58
Sharma et al.^120^	Indirect hybrid SD	Tomato	4.21
Proposed DSD	DSD integrated with a TSFPSC	STFF	1.59 years

## Conclusion, recommendations, limitations and future work

The current study aimed to develop an indirect forced solar dryer integrated with a three-sided flat plate solar collector (TSFPSC). This design was intended to maximize sunlight collection, particularly during the morning and afternoon, thereby enhancing the thermal efficiency of solar collectors compared to fixed flat plate models. The developed solar dryer was utilized to dry salted tilapia fish fillets (STFF) at three slice thicknesses: 4 mm, 8 mm, and 12 mm, using hot air at a consistent speed of 0.5 m/s. The obtained results indicated that using DSD led to a reduction in drying time of about 53.3% and 61.11% compared to the DSD. The EMD of different STFF samples in both drying systems ranged from 0.51 × 10^–10^ to 9.16 × 10^–10^ m^2^/s. Furthermore, all eleven basic TLDM used in the current study could be used to predict the drying behavior of STFF in the drying process, while the Combined Two-Term and Page model had the best fitting for the OSD system, and the Modified Midilli II model and Combined Two-Term and Page model had the best fitting for the DSD system. In terms of economic analysis, the annual capital cost and annualized investment cost were found to be 22.458 and 21.334 USD, respectively. Additionally, the EP period was 1.59 years, and the net CO_2_ mitigation over the DSD lifetime was 14 tons.

### Recommendations

The combined Two-Term and Page models are the best TLDM for describing the drying behavior of the OSD across all fillet thicknesses. In contrast, the modified Midilli II model is the most effective TLDM for characterizing the drying behavior of the DSD for fillet thicknesses of 4 mm and 8 mm. Finally, the combined Two-Term and Page model is also the best TLDM for describing the drying behavior of the DSD at a fillet thickness of 12 mm.

### Future work

The integration of artificial intelligence, machine learning, and PVT drying can potentially transform solar dryers in the forthcoming years. This advancement would enhance their intelligence, efficiency, and user-friendliness. Moreover, applying artificial neural network (ANN) modeling can facilitate a comprehensive analysis of the data obtained.

## Data Availability

All data are presented within the article.

## Abbreviations

TSFPSC: Three-sided flat plate solar collector
ATO: Annual thermal outputs
STFF: Salted tilapia fish fillets
OSD: Open sun drying
DSD: Developed solar dryer
TLDM: Thin-layer drying models
MC: Moisture content
EMD: Effective moisture diffusivity
EE: Embodied energy
TFF: Tilapia fish fillets
EP: Energy payback
d.b.: Dry base
w.b.: Wet base
DR: Drying rate
TE: Thermal efficiency
SEC: Specific energy consumed
Mr: Moisture removal
MR: Moisture ratio
